# Role of LTA4H Polymorphism in Tuberculosis-Associated Immune Reconstitution Inflammatory Syndrome Occurrence and Clinical Severity in Patients Infected with HIV

**DOI:** 10.1371/journal.pone.0163298

**Published:** 2016-09-19

**Authors:** Gopalan Narendran, Dhanasekaran Kavitha, Ramesh Karunaianantham, Leonardo Gil-Santana, Jilson L. Almeida-Junior, Sirasanambatti Devarajulu Reddy, Marimuthu Makesh Kumar, Haribabu Hemalatha, Nagesh Nalini Jayanthi, Narayanan Ravichandran, Raja Krishnaraja, Angamuthu Prabhakar, Tamizhselvan Manoharan, Lokeswaran Nithyananthan, Gunasundari Arjunan, Mohan Natrajan, Soumya Swaminathan, Bruno B. Andrade

**Affiliations:** 1 National Institute for Research in Tuberculosis, Chennai, India; 2 Unidade de Medicina Investigativa, Laboratótio Integrado de Microbiologia e Imunorregulação, Centro de Pesquisas Gonçalo Moniz, Fundação Oswaldo Cruz, Salvador, Brazil; 3 Multinational Organization Network Sponsoring Translational and Epidemiological Research (MONSTER) Initiative, Fundação José Silveira, Salvador, Brazil; 4 Curso de Medicina, Faculdade de Tecnologia e Ciências, Salvador, Brazil; 5 Government Hospital of Thoracic Medicine, Tambaram Sanatorium, Chennai, India; 6 Government Vellore Medical College and Hospital, Vellore, India; 7 India Council of Medical Research, Health secretary, Department of Health Research, Ministry of Health and Family Welfare, New Delhi, India; Rush University, UNITED STATES

## Abstract

**Background:**

Paradoxical tuberculosis-associated immune reconstitution inflammatory syndrome (TB-IRIS) is an inflammatory phenomenon complicating HIV management in coincidental tuberculosis (TB) infection, upon immune reconstitution driven by antiretroviral therapy (ART). Leukotriene A4 hydroxylase (LTA4H), an enzyme which converts LTA_4_ to LTB_4_, regulates the balance between the anti-inflammatory lipoxins and pro-inflammatory LTB_4_, with direct implications in TB-driven inflammation. In humans, a single nucleotide polymorphism (SNP) in the *LTA4H* promoter which regulates its transcriptional activity (rs17525495) has been identified and described to impact clinical severity of TB presentation and response to corticosteroid therapy. Notably, the role of *LTA4H* on TB-IRIS has not been previously evaluated. Here, we performed an exploratory investigation testing the association of *LTA4H* polymorphism with respect to frequency of TB-IRIS occurrence and severity of TB-IRIS presentation in HIV-TB co-infected individuals.

**Methods:**

Genotypic evaluation of the LTA4H enzyme from available samples was retrospectively correlated with clinical data captured in case sheets including IRIS details. The cohort included patients recruited from a prospective cohort study nested within a randomized clinical trial (NCT0933790) of ART-naïve HIV+ patients with newly diagnosed rifampicin sensitive pulmonary TB in South India. Frequency of the wild type genotype (CC), as well as of the mutant genotypes (CT or TT) in the IRIS and non-IRIS patients was estimated. Comparative analyses were performed between wild genotype (CC) and the mutant genotypes (CT or TT) and tested for association between the *LTA4H* polymorphisms and IRIS incidence and clinical severity.

**Results:**

A total of 142 eligible ART-naïve patients were included in the analyses. Eighty-six individuals exhibited the wild genotype (CC) while 56 had mutant genotypes (43-CT and only 13-TT). Variant allele frequency was 0.23 and 0.26 in non-IRIS group and in IRIS group, respectively. Upon ART initiation, 51 patients developed IRIS while 91 did not. IRIS incidence was 34% and 37% in the wild (CC) and mutant type (CT/TT), respectively (p = 0.858) with a higher frequency of severe IRIS presentation in the mutant genotype group compared to the wild type genotype (p = 0.0006). A logistic regression model confirmed the association between the presence of CT/TT genotypes and occurrence of severe IRIS. Corticosteroid therapy successfully resolved IRIS in all cases irrespective of the *LTA4H* genotype.

**Conclusion:**

A higher incidence of severe IRIS among patients with mutant *LTA4H* genotypes (CT and TT) was observed compared to the wild type, despite similar IRIS incidence and immune restoration in both groups. Steroids were effective in alleviating IRIS in all the genotypes.

## Introduction

Paradoxical TB-IRIS is a complex inflammatory phenomenon complicating TB management in HIV-infected patients initiating ART. This condition is characterized by an initial temporary improvement in the patient’s condition while starting anti-TB treatment (ATT) followed subsequently by a paradoxical clinical or radiological worsening with ART initiation, despite effective virological suppression [1]. TB-IRIS is associated with considerable morbidity and mortality, leading to utilization of tertiary care facilities and specialists for efficient management of this inflammatory condition [2]. The incidence of TB-IRIS has gradually increased over the years, with renewed interest in starting ART within the intensive phase of TB treatment in patients with HIV co-infection [3,4]. Steroids are the corner stone for IRIS therapy, acting as effective suppressor of the inflammatory outburst caused by hyper activation of immune system and cytokine storm [5,6]. The host innate and adaptive immune responses, as well as mycobacterial antigen load, have been described to play critical roles in the immune pathogenesis of TB-IRIS [7,8]. Whether lipid mediators such as leukotrienes play relevant role in TB-IRIS pathogenesis is unknown.

Leukotrienes are important mediators of innate immunity in several clinical settings, including HIV infection [9]. LTA4H is a key enzyme involved in inflammatory cascades associated with arachidonic acid pathways. Activation of LTA4H ultimately influences the conversion of LTA4 to either the pro-inflammatory mediator leukotriene B4 (LTB4) or to the anti-inflammatory lipoxin A4 (LXA4) [10,11]. The balance between LTB4 and LXA4 in hosts infected with mycobacteria seems to influence immunopathology. In experimental models of mycobacterial infection, both the excessive or deficient LTA4H enzymatic activity directly impact susceptibility to severe disease [10,11]. Abnormal increases in LTB4 production are associated with excessive inflammation and in such scenario the disease is caused mostly by immunopathology [10,11]. On the converse, deficient LTA4H activity leading to increased LXA4 production is associated with down-modulation of immune responses which are essential to control mycobacterial replication inside host cells [10,11]; in which case, disease is caused by a failure to restrain the pathogen growth [11].

Recently, a single nucleotide polymorphism (SNP), namely rs17525495 (C/T), located very close to the promoter site of *LTA4H*, was described to regulate LTA4H activity in humans [10]. This polymorphism, consisting of the wild type being CC and the mutant type being CT and TT, respectively, has been shown to directly influence inflammatory responses [10]. Notably, the double substituted mutant (TT) genotype exhibits higher transcriptional activity of *LTA4H* leading to higher levels of LTB4. In this setting, LTB4 chemo attracts neutrophils, monocytes and eosinophils, stimulating production of a variety of proinflammatory cytokines and mediators, augmenting pathological inflammation [10]. On the converse, the CC genotype is associated with lower levels of LTA4H and diminished production of LTB4 (and increased LXA4) with paucity of inflammation compromising containment of infection leading to progression and dissemination [10]. In a well-established model of mycobacterial infection of zebrafish, both homozygous genotypes CC and TT were associated with increased susceptibility to infection either due to failure of innate responses to control bacterial growth or excessive inflammation leading to tissue damage [10]. Importantly, in a cohort of TB meningitis patients, the authors inferred that disease severity and mortality were high in individuals with homozygous alleles while heterozygosity conferred clinical protection. This discovery had applied pharmaco-therapeutic significance, as only patients presenting with the TT genotype benefitted by adjuvant steroid therapy [10].

Intriguingly, no genetic determinants driving susceptibility to TB-IRIS occurrence (predisposing to inflammation) or disease severity has yet been identified. In the present study, we investigated the role of *LTA4H* SNPs in influencing the incidence or clinical severity of confirmed cases of TB-IRIS.

## Material and Methods

### Ethics Statement

All clinical investigations were conducted according to the principles expressed in the Declaration of Helsinki. Written informed consent was obtained from each participant at the study enrollment. All materials given to the research team were de-identified. The study was approved by the Scientific Advisory Committee and Institutional Ethics Committee of the National Institute for Research in Tuberculosis (Chennai) and is registered on clinicaltrials.gov (NCT 933790).

### Study Design

Genomic DNA was extracted using QIAamp DNA blood mini kit (Qiagen, Hilden, Germany) and quantitated on Thermo Fisher’s Nano Drop 2000 spectrophotometer (NanoDrop Technologies Inc., Wilmington, DE, USA), from available blood samples of those patients who had consented in the TB-IRIS study, for use of their biological samples for future analysis. *LTA4H* gene promoter region SNP (rs17525495) was analyzed from extracted DNA by using Taqman SNP genotyping assay by 7500 Real-Time PCR System (Applied Biosystems, USA) and Sequence Detection Software (SDS) v1.3.1. The frequency of occurrence of the three genotypes namely CC, CT and TT in the IRIS patients and Non-IRIS controls were evaluated. The TB-IRIS sub-study was a nested study within the randomized clinical trial (NCT0933790) [12] at the National Institute for Research in Tuberculosis (NIRT), Chennai, India.

Data was collected retrospectively from the study participants in the TB-IRIS study. Eligibility criteria included age 18 years or above, with newly diagnosed culture confirmed rifampicin sensitive pulmonary TB and advanced HIV disease, being ATT and ART naïve at enrollment. Overall characteristics of the study participants are shown in Table 1. These patients had been prospectively followed for occurrence of IRIS after ATT and ART initiation. Diagnosis and severity of IRIS was confirmed by a panel of independent physicians (N.J., N.R., K.R., S.S., P.A.), using modified INSHI criteria [1] which included a viral log decline of at least 0.5 log_10_ copies/ml with cultures being negative for *Mycobacterium tuberculosis* at IRIS event, as described previously [3].

**Table 1:** Characteristics of the patients IRIS and non-IRIS at pre-ART.

Characteristic	IRIS	Non-IRIS	p-value
**N**	51	91	
Age, median years (IQR)	36 (32–42)	39 (33–45)	0.141
Male, no. (%)	44 (86.2)	67 (73.6)	0.093
Weight, median Kg (IQR)	38 (43–50)	45 (39–49)	0.988
Time to ART, median days (IQR)	19 (10–30)	17 (3.5–47.5)	0.944
***Hematology (pre-ART)–median (IQR)***			
Hemoglobin, g/dL	8.8 (7.6–10.2)	10.2 (8.7–11.8)	0.0004
Hematocrit, %	43 (38–50)	45 (39–49)	0.989
RBC count, x10^6^/mL	3.4 (2.8–3.9)	3.8 (3.1–4.2)	0.022
CD4+ T-cells/mL	89 (40–163)	160 (81.5–299.5)	0.0001
CD8+ T-cells/mL	591 (288–1022)	724.5 (398.5–1229)	0.139
CD4/CD8 ratio	0.13 (0.08–0.27)	0.23 (0.16–0.41)	0.0006
HIV RNA, log10 copies/mL plasma	5.69 (5.29–5.87)	5.15 (4.31–5.65)	<0.0001
***TB evaluation (pre-ART)***			
Sputum smear grade–no. (%)			0.444
0	4 (7.8%)	4 (4.4%)	
1+	24 (47%)	43 (47.2%)	
2+	16 (31.3%)	23 (25.2%)	
3+	6 (11.7%)	19 (20.8%)	
Sputum culture grade–no. (%)			0.0267
1+	13 (26.5%)	29 (33.7%)	
2+	18 (36.7%)	14 (16.3%)	
3+	18 (36.7%)	43 (50%)	
Presence of military TB–no. (%)	4 (7.8%)	9 (9.9%)	0.770
***Parameters at time of IRIS event or equivalent timepoint–median (IQR)***			
CD4+ T-cells/mL	160.5 (102.3–304.3)	267 (160.5–407.5)	0.002
CD8+ T-cells/mL	608 (371–907.8)	860.5 (542.5–1236)	0.0006
CD4/CD8 ratio	0.35 (0.19–0.49)	0.35 (0.22–0.49)	0.647
HIV RNA, log10 copies/mL plasma	3.12 (2.60–3.58)	3.13 (2.59–4.57)	0.582
Hemoglobin, g/dL	9.7 (8.4–10.9)	11.1 (9.8–12.2)	<0.0001
***Change in hematological parameters at the time of IRIS or equivalent period from baseline–median (IQR)***			
CD4+ T-cells	82 (6–166)	67 (17–141.5)	0.468
CD8+ T-cells	-17 (-200–180)	85 (-115–329)	0.045
CD4/CD8 ratio	0.11 (0.02–0.37)	0.06 (-0.01–0.14)	0.016
HIV RNA	-5.66 (-5.87–-5.24)	-5.52 (-4.75–2.28)	<0.0001
Hemoglobin	0.45 (-0.62–1.9)	0.6 (-0.5–2.0)	0.643
***LTA4H genotypes*–no. (%)**			0.858
Wild type (CC)	30 (58.8)	56 (61.5)	
Mutant type (CT or TT)	21 (41.2)	35 (38.5)	

Continuous variables were compared using the Mann-Whitney *U* test. Frequencies of smear and culture grades were compared using the chi-square test. Frequencies of military TB were compared using the Fisher’s exact test. Changes from baseline were calculated subtracting values observed at pre-ART from those detected at time of IRIS or equivalent timepoint.

Detailed data on clinical history and physical examination was available as patients had their ATT administered for 6 months under direct supervision and the research staff could actively screen for IRIS every time they visited the clinic. Results of immunological parameters like CD4+ and CD8+ T-cell counts, CD4/CD8 ratio, as well as hemoglobin, hematocrit and viral load levels done at baseline, IRIS, 2–3 months after ART in Non-IRIS and at the end of ATT that were sequentially collected were compared and correlated with the genotype of *LTA4H*. R.K. and H.H., the concerned lab personnel elucidating the genotype were blinded to the clinical history and IRIS classification. Similarly, the independent panel did not have access to *LTAH4* gene polymorphism data which was generated from stored samples retrospectively. Both these data were later complied and analyzed by B.B.A., D.K., S.S., and L.G-S.

Patients experiencing IRIS were initially treated with a 3–5 day course of non-steroidal anti-inflammatory drugs based on severity, after which steroids were instituted in the dosage of 0.5–2 mg/kg and tapered over a 4–8 week period depending on the response. Presentation of symptoms and signs of IRIS in patients were clinically classified further into mild and severe cases. Severity was based on either a Karnofsky scale of 50 or less, or clinical condition mandating admission or prolongation of hospital admission [6,12,13]. These evaluations were performed by an independent panel of physicians (N.J., N.R., K.R., S.S., P.A.).

### Statistical analysis

Statistical analysis was carried out using the SPSS 20.0 (IBM statistics), Graphpad Prism 6.0 (GraphPad Software, San Diego, CA) and JMP 11.0 (SAS Institute Inc., USA) software taking the wild genotype (CC) as control and the combined mutant genotypes (CT and TT) as cases. The Pearson’s chi-square test was performed to check whether the observed genotype frequencies are consistent with Hardy-Weinberg equilibrium. Median values with interquartile ranges (IQR) were used as measures of central tendency. The Fisher’s exact test was used to compare frequency data (nominal variables). Continuous variables were compared between the study groups using the Mann-Whitney *U* test. A logistic regression analysis to test associations with the occurrence of severe IRIS was employed utilizing pre-ART characteristics, which presents a p-value below 0.5 (arbitrary cut-off) in univariate analyses comparing severe vs. non-severe IRIS. Odds ratios were adjusted for all variables in the model. All the analyses were pre-specified. A p-value less than 0.05 was considered statistically significant.

## Results

Of 142 ART naïve TB-HIV co-infected patients included in this study, 51 (35.9%) had developed TB-IRIS upon ART initiation (Table 1). At the study enrollment (pre-ART), individuals who developed IRIS during the study follow up were similar to non-IRIS patients with regard to age, gender, days from implementation of anti-TB treatment to ART initiation (time to ART) and frequency of extra-pulmonary TB (Table 1). As expected from previous reports from India [3,8], patients who experienced IRIS had a lower pre-ART CD4+ T-cell and red blood cell counts as well as hemoglobin levels, while exhibiting higher viral loads in plasma than non-IRIS counterparts (Table 1). In addition, we observed an increased frequency of patients presenting with higher *M*. *tuberculosis* sputum culture grades at the time of study enrollment in the TB-IRIS group compared to non-IRIS (p = 0.0267, Table 1).

At the time of IRIS event, patients still exhibited lower CD4+ and CD8+ T-cell counts as well as hemoglobin concentrations than those who did not developed IRIS (Table 1). Changes in CD4/CD8 ratio and HIV viral load values from the study baseline were more pronounced in TB-IRIS patients than those who had uneventful immune recovery during follow up (Table 1). We next compared changes in values of hematological parameters from pre-ART to the time of IRIS event or equivalent time between TB-IRIS and non-IRIS groups. Following this approach, although we observed no significant difference in CD4+ T-cell count changes between IRIS and non-IRIS patients, there was a more substantial decrease in HIV viral loads in plasma in IRIS individuals than in those who did not develop this inflammatory syndrome upon ART initiation (Table 1).

Genotyping of *LTA4H* revealed that within the entire study population (142), 86 individuals had the wild genotype (CC) while 56 had the mutant genotype (43—CT and 13—TT). Of the 51 IRIS patients, 58.8% (30/51) had the wild type while 41.2% (21/51) had the mutant type of *LTA4H*. In the non-IRIS group comprising of 91 patients, 61.5% (56/91) had the wild type compared to 38.5% (35/91) having the mutant type (Table 1). The frequency of mutant genotypes (CT or TT) in the group of patients who develop IRIS was not statistically different than that observed in the group of individuals from the non-IRIS group (Table 1). Variant allele frequency was 0.23 and 0.26 in the non-IRIS group and in the IRIS group, respectively. In both the groups, the observed genotypic frequencies were consistent with Hardy-Weinberg equilibrium.

Moreover, evaluation of clinical severity was performed within the IRIS groups using case records. Out of 51 TB-IRIS patients, 29 (56.9%) presented with severe IRIS events. Median time interval between ATT and ART was 21.5 days (IQR: 9.7–31) in non-severe IRIS cases and 16 days (IQR: 10–28) in severe cases (p = 0.647, Table 2). On average, time from ART initiation to IRIS onset was similar between severe IRIS (median: 13 days, IQR: 7–21) and non-severe IRIS cases (median 19 days, IQR: 10–46, p = 0.057, Table 2). At pre-ART and also at the time of IRIS event, individuals who would present with severe IRIS were indistinguishable from those of milder cases at the time of IRIS event with regard to clinical, hematological and microbiological parameters (Table 2).

**Table 2:** Comparison between severe and non-severe cases of patients in the TB-IRIS study.

Characteristic	Severe IRIS	Non-severe IRIS	p-value
**N**	**29**	**22**	
Age, median years (IQR)	37 (34.5–46)	36 (29.2–40)	0.240
Male, no. (%)	26 (89.7)	18 (81.8)	0.447
Weight, median Kg (IQR)	42 (38–48)	44 (38–55)	0.469
Time to ART, median days (IQR)	16 (10–28)	21.5 (9.7–31)	0.647
Time from ART to IRIS, median days (IQR)	13 (7–21)	19 (10–46)	0.057
***Hematology (pre-ART)–median (IQR)***			
Hemoglobin, g/dL	8.9 (7.5–10.4)	8.7 (7.8–9.8)	0.641
Hematocrit, %	42 (38–48)	44.5 (37.5–55.2)	0.475
RBC count, x10^6^/mL	3.4 (2.7–4.1)	3.4 (2.83.7)	0.696
CD4+ T-cells/mL	89 (39.5–168)	84 (41.5–141.8)	0.849
CD8+ T-cells/mL	239 (242.5–1095)	589.5 (309–958.3)	0.992
CD4/CD8 ratio	0.15 (0.07–0.27)	0.11 (0.08–0.27)	0.886
HIV RNA, log10 copies/mL plasma	5.79 (5.39–5.87)	5.60 (5.17–5.87)	0.562
***TB evaluation (pre-ART)***			
Sputum smear grade–no. (%)			0.7006
0	2 (6.8%)	1 (4.5%)	
1+	14 (48.2%)	10 (45.5%)	
2+	9 (31%)	7 (31.8%)	
3+	2 (6.8%)	4 (18.2%)	
Sputum culture grade–no. (%)			0.815
1+	8 (27.5%)	5 (22.7%)	
2+	9 (31%)	9 (40.9%)	
3+	10 (34.5%)	8 (36.3%)	
Presence of miliary TB–no. (%)	2 (6.8%)	2 (9%)	1.0
***Parameters at time of IRIS event or equivalent timepoint–median (IQR)***			
CD4+ T-cells/mL	149 (95.5–287)	210 (127.5–333.5)	0.254
CD8+ T-cells/mL	490 (352–915)	687 (368.5–890.5)	0.768
CD4/CD8 ratio	0.30 (0.16–0.48)	0.40 (0.21–0.53)	0.340
HIV RNA, log10 copies/mL plasma	3.24 (2.60–3.78)	2.85 (2.60–3.38)	0.105
Hemoglobin, g/dL	8.9 (8.3–10.6)	10 (8.8–11.4)	0.145
***Change in hematological parameters at the time of IRIS or equivalent period from baseline–median (IQR)***			
CD4+ T-cells	71 (2.5–150)	122 (36.7–192)	0.123
CD8+ T-cells	-7 (-382–273)	-19.5 (-145.5–141)	0.856
CD4/CD8 ratio	0.06 (0.0–0.3)	0.26 (0.02–0.42)	0.189
HIV RNA	-5.66 (-5.87–-5.37)	-5.65 (-5.87–-5.14)	0.991
Hemoglobin	0.25 (-0.87–1.6)	1.0 (0.1–2.5)	0.067
***LTA4H genotypes*–no. (%)**			0.0006
Wild type (CC)	11 (37.9)	19 (86.4)	
Mutant type (CT or TT)	18 (62.1)	3 (13.6)	

Continuous variables were compared using the Mann-Whitney *U* test. Frequencies of smear and culture grades were compared using the chi-square test. Frequencies of military TB were compared using the Fisher’s exact test. Changes from baseline were calculated subtracting values observed at pre-ART from those detected at time of IRIS or equivalent timepoint.

Of note, our data revealed an increased frequency of individuals with mutant *LTA4H* genotypes (CT or TT) in the severe IRIS group (62%, 18/29), compared to that detected in in the group of non-severe IRIS patients 13.6%, 3/22, p = 0.0006, Table 2). These findings argue that presence of mutant *LTA4H* genotypes is associated with occurrence of severe TB-IRIS upon ART initiation. To better test this hypothesis, we employed a multivariate logistic regression model designed to examine the odds of severe IRIS compared to non-IRIS, including all parameters which exhibited p-values below 0.5 in univariate comparisons between these clinical groups (age, gender, weight, time from ART to IRIS and hematocrit level. Aside from presence of mutant *LTA4H* genotypes). Our results demonstrated that presence of mutant *LTA4H* genotypes was independently associated with increased odds of severe TB-IRIS (adjusted odds ratio: 15.33, IQR: 2.81–63.75, p = 0.002, Table 3).

**Table 3:** Associations between pre-ART characteristics and occurrence of severe IRIS.

Characteristic	Unadjusted OR (95% CI)	p-value	Adjusted OR (95% CI)	p-value
Age	0.94 (0.87–1.02)	0.125	0.92 (0.32–1.02)	0.105
Male gender	0.99 (0.99–4.94)	0.987	0.83 (0.11–6.10)	0.834
Weight	1.04 (0.97–1.11)	0.264	1.12 (0.91–1.24)	0.089
Time from ART to IRIS	1.56 (0.99–2.01)	0.068	1.32 (0.68–2.33)	0.085
Hematocrit level	1.08 (0.95–1.92)	0.274	1.05 (0.97–1.18)	0.268
Mutant *LTA4H* genotype (CT or TT)	10.36 (2.48–43.31)	0.001	15.33 (2.81–63.75)	0.002

Logistic regression model was used to test associations with parameters assessed at pre-ART from Table 2 which exhibited p-values below 0.5 for comparison between severe and non-severe IRIS. Adjustment was performed for all parameters presenter. CI, confidence interval; OR, odds ratio.

## Discussion

To our knowledge, this is the first study in IRIS parlance to look at the influence of *LTA4H* polymorphism on TB-IRIS occurrence and severity of clinical presentation. We found similar incidence of IRIS in both the wild type and the mutant genotype type for the LTA4H enzyme. Interestingly, there was a substantially high frequency of the mutant genotypes of *LTA4H* (CT and TT) in severe TB-IRIS cases compared to that observed in mild IRIS cases. The ATT-ART time interval in both these clinical groups were comparable with equivalent reduction in viral load after ART initiation. Considering that the immune recovery and ATT-ART interval were similar between severe and non-severe IRIS cases, with equivalent decline in viral loads, the increased frequency of mutant *LTA4H* genotypes in the group of severe IRIS argues that the genotypic polymorphism involving *LTA4H* could be a possible cause for exuberant inflammation; translating into a higher cytokine immune outburst in the CT/ TT type, in agreement with previous studies [9,10].

In the study on role of *LTA4H* polymorphism in TB meningitis among Vietnamese patients by Tobin et al., there were two peaks of higher mortality corresponding to the homozygous wild type CC (with susceptibility to the disease) and the homozygous mutant type TT (hyper-inflammatory tendency), with the heterozygous group (CT) offering the best survival advantage. In this setting, the higher inflammatory response in the TT type contributed to higher mortality but also derived maximum benefits with steroid use. In our study, this bimodal pattern of morbidity was absent. This difference in the effect of *LTA4H* polymorphism could be contributed by various factors. Better LTA4H production in the CC type after immune restitution prevented disease susceptibility [8]. Different mechanisms could operate segregating IRIS and meningitis, or there could be ethnic variation between the Vietnamese and the South Indian population in the two cohorts analyzed. Since, inflammation was less in wild type (CC) individuals, it had resulted in uneventful immune recovery or milder form of IRIS in this study group. On the contrary, the mutant genotype (CT/TT) had 21 times more incidence of severe IRIS compared to the wild type.

Tobin et al. study demonstrated that only a subset of patients (the homozygous mutant TT) benefitted by the use of steroids in TB meningitis. Steroids proved detrimental in the immunocompromised group probably due to unchecked progression of infection. However, in our study, all of IRIS patients, who exhibit disease primarily by dysregulation of inflammation due to cytokine storm, favorably responded to steroids [5]. More recently, the inflammatory mechanisms involved in TB-IRIS have been better defined by means of identification of myeloid cell types involved [8] as well as the involvement of inflammasome activation [14–16]. It is possible that the inflammasome activation may result in increased production of eicosanoids, resulting in an “eicosanoid storm” [17], which may be as relevant as a cytokine storm in the context of TB-IRIS.

It is possible that the smaller sample of only 31 IRIS cases within the 86 patients with CC genotype could be the reason for not seeing observable differences in the steroid response based on *LTA4H* genotype. The beneficial role of steroids in IRIS therapy has been established by the Meintjes et al. [6,18]. Usefulness of steroids has also been established in a study by Tadokera et al. which demonstrated prospectively the rise in expression of several inflammatory biomarkers during TB-IRIS event and the subsequent fall of these parameters in serum with steroid administration that respectively coincided clinically with the appearance and disappearance of symptoms and signs in IRIS [18,19]. In this study, the response to steroids was shown to be mediated through suppression of predominately pro-inflammatory cytokine responses of innate immune origin and not via reduction in the number of antigen-specific T cells in the peripheral blood as usually expected [18]. It is possible that leukotrienes such as LTB4 play an important role in TB-IRIS as these lipid mediators have been described to play a critical role in innate immune networks [9].

Marais et al. have previously examined paradoxical TB-IRIS at the meningeal site, looking at the combined phenomenon of TB-IRIS and TB meningitis. The authors demonstrated that a relatively higher dose of steroids may be required in TB meningitis (0.75–1.5 gm/kg) for maximizing response [20]. Hence, it is possible that a higher dose could overcome the inflammation even in the mutant type provided immunity is effectively restored, which deserves further exploration.

In our cohort, systemic concentrations of inflammatory mediators derived from the innate system, such as CRP and IL-6, were found to be elevated in TB-IRIS patients both before ART and at IRIS and served as good candidate biomarkers for IRIS prediction and prognosis [3]. Strengths of our study were that the baseline parameters in the two genotypic groups of *LTA4H* were comparable with respect to immunodeficiency status. Both the wild type and the mutant genotype groups had ART initiation at the same time apart from similar immune recovery after ART initiation. In addition, our study population was strictly a cohort of rifampicin sensitive culture positive pulmonary TB, which brings advantages in ascertaining the diagnosis of IRIS. Treatment of ATT and ART were under strict supervision ensuring adherence to both classes of drugs. Bias had been minimized by the lab personnel who were blinded to IRIS status and severity while evaluating the *LTA4H* polymorphism and the independent panel which certified the patients as IRIS and Non-IRIS was unaware of the *LTA4H* polymorphism in the patient.

The limitations of our study were that the sample size was small to elucidate the selective use of steroids / restricted benefit derived in a particular group. The estimation of immunological parameters and fixation of the second time point among controls (non-IRIS patients) i.e. ATT-ART interval was variable. However, the Non-IRIS group did not experience IRIS subsequently for at least a year of follow-up after ATT completion and remained uneventful. Finally, TB-IRIS incidence in our study was approximately 36%, higher than most of other studies. We speculate that this discrepancy occurred because our clinical protocol included primarily patients with positive sputum culture, rifampicin sensitive pulmonary TB with advanced HIV being initiated concomitantly on ATT and ART and prospectively monitored for IRIS occurrence. Supervision was done every day as part of DOTS (Directly observed therapy) strategy for ATT administration and active surveillance may have also contributed to a higher detection rate of IRIS.

Our findings are important in taking precautionary measures in the management of IRIS where a genotypic test could help in predicting severity and could potentially guide management of steroid dosage. We concur with the findings of Tobin et al. [10,11] that the *LTA4H* genotype could play a crucial role in the inflammatory reactions of TB including IRIS. The use of steroids in IRIS irrespective of the genotype is beneficial and clinically relevant with the incidence of IRIS being more in resource limited settings where patients enroll in the ART program in the advanced stage of disease.
